# The Impact of Extra-Domain Structures and Post-Translational Modifications in the Folding/Misfolding Behaviour of the Third PDZ Domain of MAGUK Neuronal Protein PSD-95

**DOI:** 10.1371/journal.pone.0098124

**Published:** 2014-05-20

**Authors:** Javier Murciano-Calles, Marta Marin-Argany, Eva S. Cobos, Sandra Villegas, Jose C. Martinez

**Affiliations:** 1 Departamento de Química Física e Instituto de Biotecnología, Facultad de Ciencias, Universidad de Granada, Granada, Spain; 2 Departament de Bioquímica i Biología Molecular, Facultat de Biociències, Universitat Autónoma de Barcelona, Barcelona, Spain; Harvard Medical School, United States of America

## Abstract

The modulation of binding affinities and specificities by post-translational modifications located out from the binding pocket of the third PDZ domain of PSD-95 (PDZ3) has been reported recently. It is achieved through an intra-domain electrostatic network involving some charged residues in the β2–β3 loop (were a succinimide modification occurs), the α3 helix (an extra-structural element that links the PDZ3 domain with the following SH3 domain in PSD-95, and contains the phosphorylation target Tyr397), and the ligand peptide. Here, we have investigated the main structural and thermodynamic aspects that these structural elements and their related post-translational modifications display in the folding/misfolding pathway of PDZ3 by means of site-directed mutagenesis combined with calorimetry and spectroscopy. We have found that, although all the assayed mutations generate proteins more prone to aggregation than the wild-type PDZ3, those directly affecting the α3 helix, like the E401R substitution or the truncation of the whole α3 helix, increase the population of the DSC-detected intermediate state and the misfolding kinetics, by organizing the supramacromolecular structures at the expense of the two β-sheets present in the PDZ3 fold. However, those mutations affecting the β2–β3 loop, included into the prone-to-aggregation region composed by a single β-sheet comprising β2 to β4 chains, stabilize the trimeric intermediate previously shown in the wild-type PDZ3 and slow-down aggregation, also making it partly reversible. These results strongly suggest that the α3 helix protects to some extent the PDZ3 domain core from misfolding. This might well constitute the first example where an extra-element, intended to link the PDZ3 domain to the following SH3 in PSD-95 and in other members of the MAGUK family, not only regulates the binding abilities of this domain but it also protects PDZ3 from misfolding and aggregation. The influence of the post-translational modifications in this regulatory mechanism is also discussed.

## Introduction

Based on high-throughput experimental and computational approaches it has been described that interactomes organize in hubs and super-hubs where a high amount of different metabolic routes join. The most typical example of such hub proteins is the membrane-associated guanilate kinase (MAGUK) family, to which PSD-95 (post-synaptic density-95 protein) belongs, constituted by three PDZ (postsynaptic density protein-95/discs large/zonula occludens-1), one SH3 and one guanilate kinase (GK) domains [1], [2], [3], [4], [5], [6]. From a functional point of view, such a hub proteins do not usually display any enzymatic activity and are arranged as multi-domain proteins, in which the domains are conformationally independent, and are interconnected by relatively short amino acid sequences. This modular arrangement confers hub proteins a high conformational plasticity, essential in multifaceted processes like signal transduction, cell adhesion or molecular trafficking, whether at neuronal synapses in the particular case of PSD-95, or at tight junctions, cell growing and division in the case of other members of this family [7]. From a dynamic point of view, hub proteins usually display a considerable structural disorder, showing highly variable regions that develop a variety of changes related to the multi-modular arrangement [8]. These regions have been identified as the loops and turns connecting secondary structures within the domains and, also, the short sequences connecting the own domains [9], [10].

The most abundant domain type in hub proteins is the PDZ, which usually recognizes C-terminal tails of a diversity of proteins, allowing these multi-modular proteins to act as scaffolds for functionally-related proteins. Nevertheless, this does not seem to be the only way of acting of PDZ domains, since a few recent studies describe some conformational aspects that can be profited by nature to develop new activities or regulate the existing ones. For example, the second PDZ domain of ZO (Zonnula occludens) proteins self-associates through swapping of the β2–β3 hairpin, driving this phenomena to the polymerization of claudins, the main event during cellular tight junctions formation [11], [12], [13].

**Figure 1:**
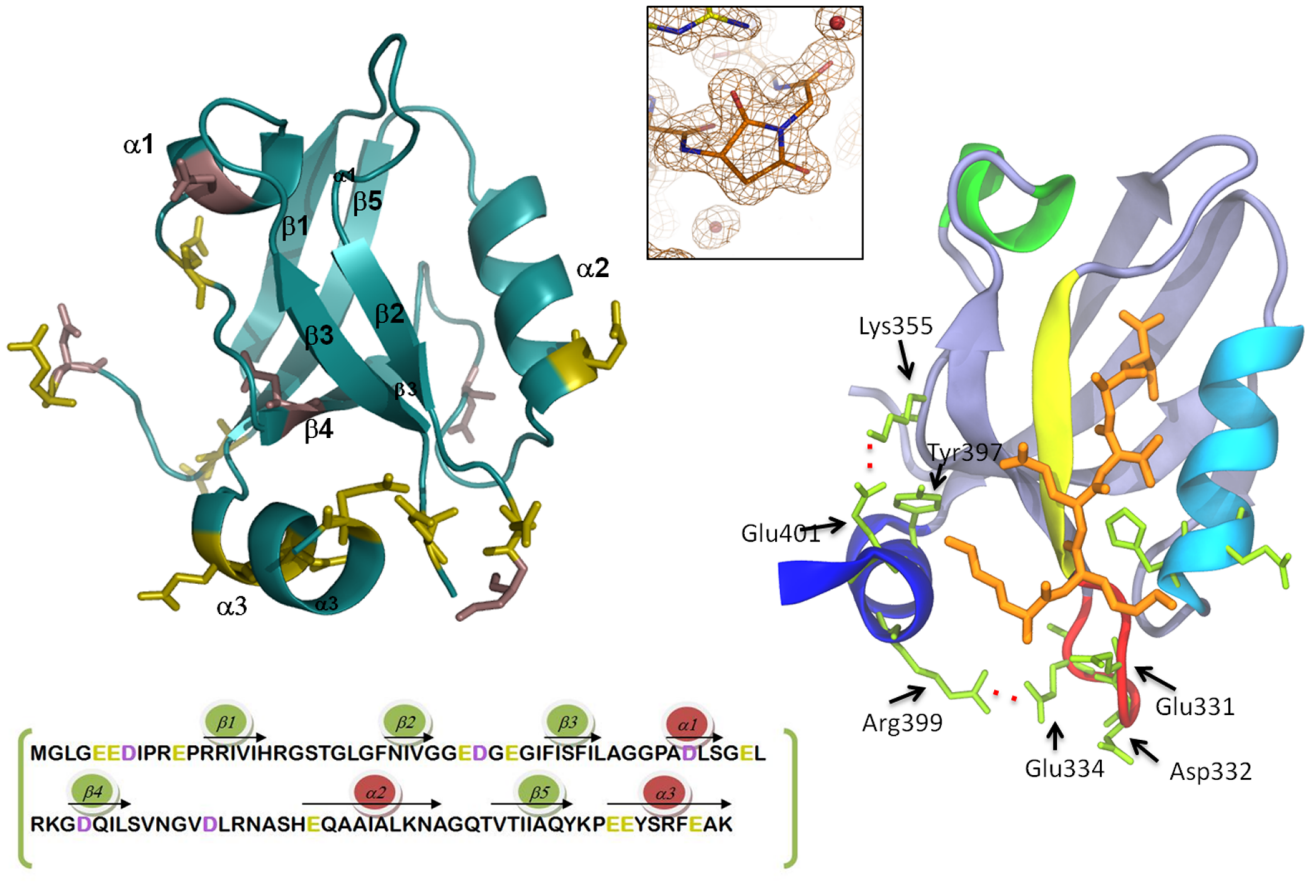
Structural details of PSD95-PDZ3 domain. Left panel: 1.4 Å X-ray structure of PSD95-PDZ3 domain (PDB code: 3K82) and the amino acids sequence showing the distribution of secondary structures. Side chains of Asp residues are coloured in purple and those of Glu residues in green. Right panel: a detail of the salt-bridges between the α3 helix (dark blue) and residues of the β2–β3 loop (red). A ligand peptide complexed with PDZ3 (PDB code: 1BE9) is shown in orange. Using the same colour code, the distribution of secondary structures along the sequence is shown below. The upper central inset shows succinimide-ring formation at Asp332 observed in the X-ray structure (PDB code: 3K82).

Structural modelling of the multi-modular organization of PSD-95 [14] shows that the whole protein develops a level of functional regulation throughout a high conformational plasticity mainly achieved by the inter-domain sequences. This structural flexibility allows, for example, the burying of some of the binding sites, domain swapping phenomena [11], or tuned changes in the specificity/promiscuity of binding. It has been proposed the term “supertertiary structure” to account for the multiplicity of conformations and states that might coexist and interchange in these multi-modular proteins under equilibrium conditions [15]. Supporting modelling, some experimental studies have shown the ability of the linker connecting PDZ1 and PDZ2 domains to develop different binding affinities and specificities in the tandem PDZ1–2 than in the isolated domains [10], [16], [17]. On the contrary, the third PDZ domain acts individually. Moreover, the modulation of binding affinities and specificities by post-translational modifications located out from the PDZ3 binding pocket through an intra-domain electrostatic network has been reported recently by us [18]. Thus, it has been reported that residue Tyr397, located at such α3 helix (Figure 1), can be phosphorylated by Src tyrosine kinase, and that the resulting phosphorylated-PDZ3 displays a lower affinity to the CRIPT peptide than PDZ3 [19] or to the KKETAV hexapeptide [18]. In addition, residue Asp332 can undergo another post-translational modification consisting in the cyclization of its side-chain after nucleophilic attack of the main-chain NH group to the CO atoms of the residue (Figure 1) [20]. This post-translational modification was found when the tridimensional structure of PDZ3 domain was solved by X-ray at high resolution. The succinimide ring participates in this intra-domain electrostatic network, and is stabilized by contacts in the crystal structure, which suggests that it is organized prior to phase changes; however, it was unstable in solution, disappearing when such a PDZ3-crystals were dissolved in buffer, showing a half-life of approximately 1 hour. This behaviour is commonly observed for succinimides found in other proteins, being described as transient modifications of Asp and Asn residues, able to affect some protein properties [20]. In fact, the negative impact of the ring upon the binding of ligand peptides by PDZ3 strongly suggests such a central role, as well as for the β2–β3 loop in the binding properties of PDZ3, where Asp332 is located [18].

Moreover, and opposite to other PDZ domains, this domain shows a C-terminal extra-helix extension, the α3 helix (Figure 1), corresponding to the amino acidic linker to the following SH3 in PSD-95. At a functional level, this extension generates an up-regulation of the affinity of PDZ3 by C-terminal protein ligands [18]; also, it mediates the interactions among PDZ3 and the following SH3 domain in PSD-95 [7]. Isothermal titration calorimetry (ITC), nuclear magnetic resonance (NMR) and molecular dynamics (MD) suggest that the salt-bridge between Glu334 of the β2–β3 loop and Arg399 of the α3 helix is the main responsible for the described regulatory mechanism by such an extra-helix, which strongly influences the interaction of a positively charged residue of the ligand peptide, usually Lys, with residues Glu331, Asp332 and Glu334 located at the β2–β3 loop [18].

Previous thermodynamic studies regarding PDZ3 conformational features have revealed that the whole domain including α3 helix unfolds under equilibrium conditions through an intermediate state that may drive to the reversible arrangement of fibrillar and annular supramacromolecular structures after incubation at temperatures where it is populated (around 60–70°C) [21]. These studies confirm the high conformational plasticity of the PDZ3 domain. We have also investigated the main molecular aspects of the intermediate state by transmission electron microscopy (TEM) and Fourier transform infrared spectroscopy (FTIR), and found that one of the two native β-sheets of PDZ3 can reorganize into the intermediate state to give rise to the fibril β-arrangement. Chemical-shift NMR analysis revealed that the β-sheet comprised by β2 to β4 strands seems to be the responsible for such a reorganization. This region is organized as a flexible β-sheet and has been predicted by different algorithms to be prone to β-aggregation [22].

Furthermore, the intermediate state does not populate at pH conditions lower than 3.5, where PDZ3 unfolds under an apparent two-state regime. This titration behaviour might be attributed to the protonation equilibriums of some Glu and/or Asp residues, the only ones whose pK_a_ values are within the range of pH 3–4 [23]. This evidence strongly pointed to some of the above mentioned Glu residues, concretely to Glu334 and Glu401, since these are the main responsible for the packing of the extra α3-helix to the domain through salt-bridges with residues Lys355 and Arg399, respectively [18]. The question still unknown is to which extent these electrostatic interactions may also determine the folding/misfolding behaviour of PDZ3, since both salt-bridges would be influenced when Glu residues protonate below pH 3, being the packing of the extra-helix to the whole PDZ3 altered.

Based on these facts and in the absence of information about the energetic and structural origins of such conformational aspects, we have analyzed here, by differential scanning calorimetry (DSC), FTIR and other spectroscopic techniques, the folding/misfolding behaviour of a truncated version of PDZ3, Δ10ct-PDZ3, where ten C-terminal residues organizing the extra-α3 helix have been removed. The Δ10ct-PDZ3 variant, a priori displaying a typical PDZ fold, is well folded although presents a bit less stability than the original PDZ3. Nevertheless, our previous evidence reveals that the unfolding behaviour appreciably changes with respect to PDZ3, being a four-state model necessary to properly describe the DSC unfolding traces [24]. To go in deep into these differences, we have performed a mutational analysis of the main salt-bridges between the domain and the extra-α3 helix. We have found that the changes observed upon unfolding are much more influenced by the long-range interaction Lys355-Glu401 rather than Glu334-Arg399. However, our previous analysis showed that the opposite is true in the case of binding to short peptides, where the latter was the almost unique responsible for the influence of the extra-α3 helix in the regulation of such interactions. Since a phosphorylation target, Tyr397, occurs at the α3 helix, our analysis may also serve to understand the influence of phosphorylation in the conformational properties of PDZ3. Finally, we have analyzed the relevance on folding/misfolding of the succinimide ring resulting from circularization of Asp332 through mutations D332G and D332P, the latter taken as an experimental approach that reasonably emulates the succinimide arrangement upon binding of short peptides to PDZ3, as previously demonstrated by calorimetry and molecular dynamics simulations [18].

## Materials and Methods

The plasmids encoding the PDZ3 mutants were derived from a wild-type PDZ3 plasmid (including residues 302–403 of the full PSD-95 protein) using the QuikChange Site-Directed Mutagenesis Kit (Agilent). The Δ10ct-PDZ3 construct (residues 302–393 of the full PSD-95 protein) was obtained by PCR amplification. All proteins were overexpressed in *Escherichia coli* BL21/DE3 and purified by standard protocols [18]. DSC, FTIR, DLS (dynamic light scattering) and fluorescence kinetics were carried out as it is referred in previous articles. Protein samples were prepared also as described elsewhere [21], [22].

## Results

### Rationale Behind Mutational Analysis of PSD95-PDZ3

In this study, we have conceived a mutational approach to understand the impact of extra-elements and post-translational modifications in PSD95-PDZ3. Thus, the extra-element evaluated was the α3 helix located at the C-terminus of the originary PDZ3 construct (residues 302–403 of PSD-95 protein). To study its contribution to the folding/misfolding of the domain we have truncated it from PDZ3 originary sequence (Δ10ct-PDZ3, residues 302–393). Mutants E334Q/L and E401R were designed to affect the main contacts between the α3 helix and the PDZ3 core. Finally, mutation D332P was designed to emulate the succinimide ring formation at residue Asp332 previously described by us [20]. Another previous study [18] has shown that Asp332 is solvent exposed at the β2–β3 loop, not contacting with any residues of the ligand or of the domain; the cyclation of this position drives to a strong modification of the conformational dynamics of the loop, being this effect the responsible of a net drop in the binding affinity of short linear peptides. These effects can be considered essentially the same when a Pro or a succinimide are present, despite the ring occurs at different positions of the backbone. Thus, within this context, we designed the mutant D332G to generate the most opposite effect to Pro from a conformational and dynamic perspective, which was confirmed by titration calorimetry and molecular dynamics [18].

### DSC Thermal Unfolding of Truncated Δ10ct-PDZ3 and E334Q/L, E401R, D332P/G Point Mutations

In previous work, we carried out DSC experiments in 50 mM potassium phosphate pH 7.5 at a heating rate of 1.5 K·min^−1^ and a set of protein concentrations ranging from 40 to 727 µM. Both, PDZ3 [21] and Δ10ct-PDZ3 [24] calorimetric traces showed two well-separated endotherms that moved opposite with protein concentration. In a three-state model this shifting indicates a higher association stoichiometry for the intermediate state with respect to both, the native and the unfolded ones. We observed a noticeable difference related to the shape of DSC profiles, since in Δ10ct-PDZ3 both transitions were sharper than in PDZ3. Additionally, reversibility decreased noticeably in Δ10ct-PDZ3, being lesser than 30%. As a consequence, the three-state unfolding model proposed to analyze PDZ3 traces (N ⇆ 1/n I_n_ ⇆ U) did not properly converge, and we decided to include the possibility of having a monomeric intermediate state under equilibrium, together with its own association process (N ⇆ I ⇆ 1/n I_n_ ⇆ U) [24]. The analysis of the populations distribution showed that, at the highest protein concentration assayed, the associated intermediate, I_n_, populated almost 100% at the temperature interval 50–90°C in the case of Δ10ct-PDZ3, whereas in the PDZ3 example it never reached more than 90% and at the narrower range of 60–80°C.

**Figure 2:**
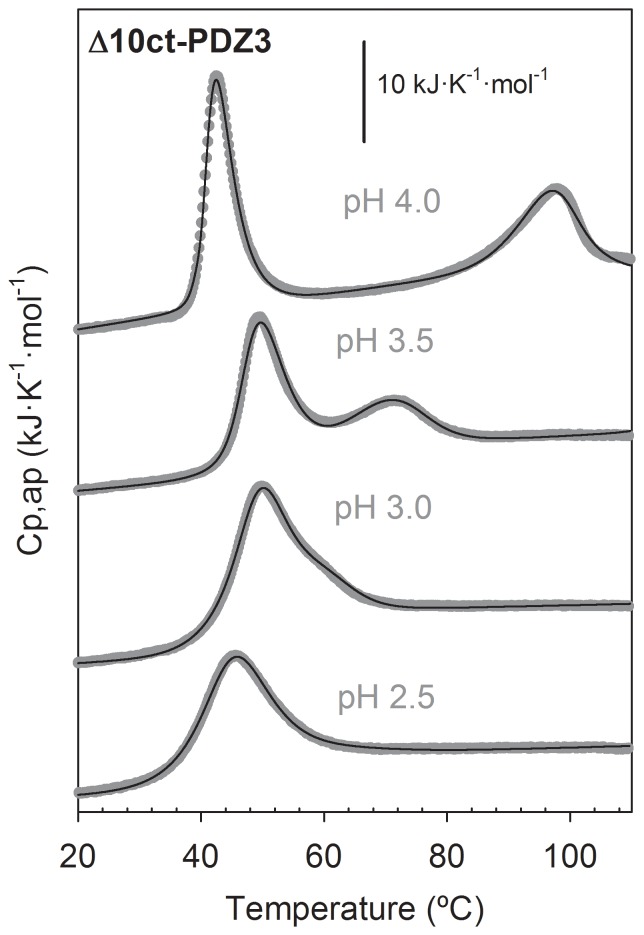
DSC thermal unfolding profiles of Δ10ct-PDZ3 as a function of pH conditions. Protein concentration was 1.3·mL^−1^ under 50 mM buffer, either acetic/acetate at pH 4.0 or glycine/HCl at other pH values. Experimental data are represented by symbols whereas solid lines through are the best fitting results obtained from respective DSC models as described in the text.

**Figure 3:**
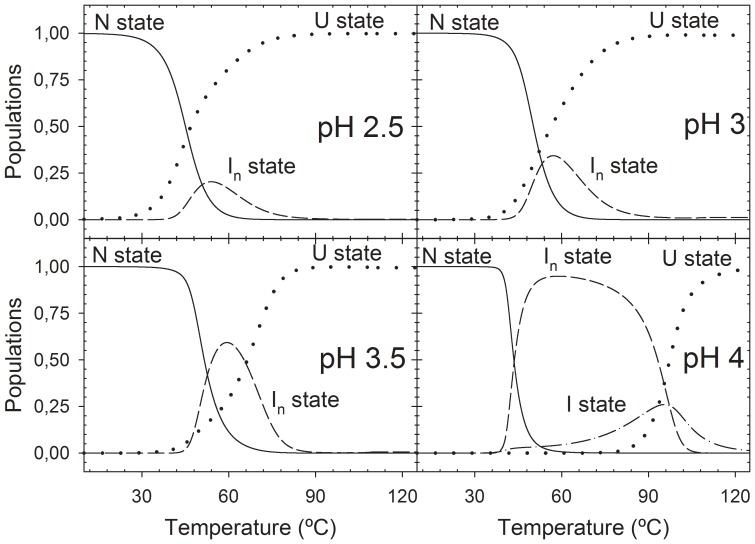
Populations analysis of the different conformational states upon thermal unfolding of Δ10ct-PDZ3 under different pH conditions. Continuous lines represent the temperature evolution of the native state and the dotted ones the respective for the unfolded state. The associated intermediate is represented by a broken line, whereas the monomeric intermediate at pH-dash line.

Since a change in the conformational equilibrium of PDZ3 below pH 3 exists [23], we have studied here the pH behaviour of the Δ10ct-PDZ3 domain by DSC. We carried out experiments at 1.3 mg·mL^−1^ protein concentration under different pH conditions; within the range 2.5–3.5 we used Glycine/HCl and at pH 4.0 acetic/acetate buffer. The behaviour is qualitatively similar to PDZ3, since at pH values 4.0–7.5 traces are biphasic (Figure 2) and at more acidic conditions both transitions approach to a single transition but, differently, we could not properly fit any of them to the two-state model as done in PDZ3 [23]. In fact, fittings in Figure 2 were performed using whether the four-state model described above for pH 4.0, or the three-state model previously described for PDZ3 for traces at lower pH values. In all of the analyses we considered a stoichiometry of n = 3 for the oligomers, as previously [21]. The thermodynamic parameters derived from the analysis are collected in Table 1. The populations distribution (Figure 3) shows that the folding intermediate destabilizes when pH drops, as it occurs in the PDZ3 case, since its relative population decreases as a whole from 100% at pH 4.0 to no more than 20% at pH 2.5, being 0% below pH 3.0 in the case of PDZ3. In addition, we observe a net influence of the reversibility of the misfolding pathway on the global reversibility of the unfolding process, having a more reversible unfolding when the intermediate is less populated. Thus, reversibility ranges from 80% at pH values 2.0–3.5 to 30% at pH 4.0–7.5. These population analyses clearly point to the conclusion that truncation of the extra-α3 helix drives to a net advantage of the associated intermediate state, since it populates in a wider temperature range and in a higher percentage than the observed when such helix is present at all of the pH conditions assayed.

**Table 1:** Thermodynamic parameters of the thermal unfolding of Δ10ct-PDZ3 domain and mutants under different acidic pH conditions in 50 mM buffer, obtained from the analysis of DSC experiments*.

N ↔ I ↔ 1/3 I_3_ ↔ U							
pH	T_N-I_ (°C)	ΔH_N-I_(T_N-I_) (kJ·mol^−1^)	T_I-In_ (°C)	ΔH_I-In_(T_I-In_) (kJ·mol^−1^)	T_I-U_ (°C)	ΔH_I-U_(T_I-U_) (kJ·mol^−1^)	ΔG_N-U_ (298) (kJ·mol^−1^)
**4.0**	53±1	250±20	106±8	−55±24	93±2	190±15	48±2

**N ↔ 1/3 I_3_ ↔ U**					
**pH**	**T_N-D_ (°C)**	**ΔH_N-D_(T_N-D_) (kJ·mol^−1^)**	**T_D-In_ (°C)**	**ΔH_D-In_(T_D-In_) (kJ·mol^−1^)**	**ΔG_N-U_ (298) (kJ·mol^−1^)**
**3.5**	56.5±0.4	233±5	73.2±0.4	−107±3	18±2
**3**	52.4±0.6	236±5	67±3	−55±9	16±2
**2.5**	45.9±0.4	196±3	57±3	−33±6	10±2
**2.5** a	44.5±0.4	200±3	–	–	10±2

*Experimental conditions were 50 mM acetic/acetate pH 4.0; 50 mM Glycine/HCl pH 2.5–3.5. The error intervals were calculated as described in the text. The values of thermodynamic magnitudes for the U⇆In and I⇆In equilibriums were estimated for a P_ref_ = 100 µM. The heat capacity functions obtained from the fittings, in kJ·K^−1^·mol^−1^ were: **pH 4:** C_pN_ = −12.8+(0.105*T); C_pU_ = −9.3+(0.084*T); C_pIn_ = −62.4+(0.233*T); C_pI_ = −35.4+(0.158*T). **pH 3.5**: C_pN_ = 2.66+(0.056*T); C_pU_ = 12.1+(0.034*T); C_pIn_ = −96.6+(0.348*T); **pH 3:** C_pN_ = 1.50+(0.059*T); C_pU_ = 15.1+(0.026*T); C_pIn_ = −11.7+(0.105*T); **pH 2.5**: C_pN_ = 10.3+(0.032*T); C_pU_ = 18.1+(0.018*T); C_pIn_ = −15.3+(0.114*T).

a Data obtained from two-state analysis.

**Figure 4:**
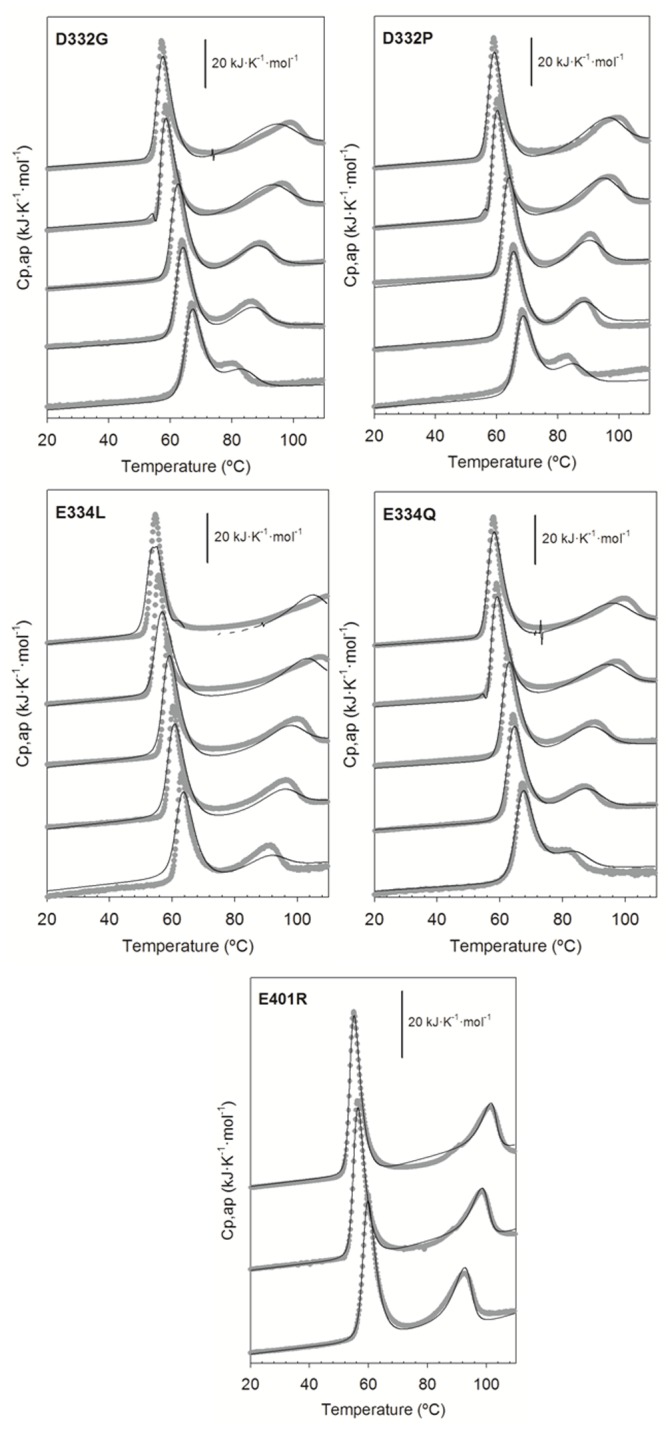
Thermal unfolding profiles of PSD95-PDZ3 mutants monitored by DSC as a function of protein concentration. Experimental conditions were 50/acetate at pH 4.0 or glycine/HCl at the other pH values. Experimental data are represented by gray symbols whereas solid lines through are the best fitting obtained from respective DSC models as described in the text.

As previously achieved for Δ10ct-PDZ3 and PDZ3, we carried out DSC experiments for every PDZ3 point mutation in 50 mM potassium phosphate pH 7.5 at a heating rate of 1.5 K·min^−1^ and a set of protein concentrations ranging from 40 to 727 µM. The calorimetric traces showed two well-separated endotherms that moved opposite with protein concentration, similarly to the described above (Figure 4). Comparing the relative shapes for a defined protein concentration, we roughly found more similarity with the previously published PDZ3 traces [21] than for the Δ10ct-PDZ3 ones [24], even showing a similar reversibility (50–60%) to the former. Consequently, we have fitted them to the three-state model used for the wild-type PDZ3, with the exception of mutant E401R. For every mutant, we assumed as common the thermodynamic parameters corresponding to the association equilibrium of the intermediate as well as the heat-capacity functions for the N, I_n_ and U states. We achieved a nice convergence, despite some of the transitions were not properly reproduced with respect to their positions in the temperature scale. The explanation would be that the association stoichiometry n = 3 derived from fittings just reflects an average of a more or less disperse distribution of misfolded species, clearly influenced by protein concentration. In any case, the traces can be well reproduced by the respective model when less restrictive individual fittings are carried out (data not shown). The enthalpies and mid-point temperatures of the other equilibriums were obtained separately for every DSC trace, being averaged later. Thus, we proceeded in the same way than previously in the case of PDZ3. The whole set of thermodynamic parameters is collected in Table 2. In the case of mutation E401R we did not achieve any reasonable convergence using the three-state approach, so we opted for the four-state one as for Δ10ct-PDZ3 considering the same restrictions. This alternative was successful, as it can be seen in Figure 4.

**Table 2:** Thermodynamic parameters of the thermal unfolding of the PSD95-PDZ3 domain and mutants in 50 mM potassium phosphate pH 7.5 obtained from the analysis of DSC experiments*.

N ↔ 1/3 I_3_ ↔ U						
	T_N-U_ (°C)	ΔH_N-U_(T_N-U_) (kJ·mol^−1^)	ΔG_N-U_(298) (kJ·mol^−1^)	T_U-In_ (°C)	ΔH_U-In_(T_U-In_) (kJ·mol^−1^)	ΔG_U-In_(343) (kJ·mol^−1^)
**PDZ3** a	70.4±0.5	335±20	39±6	79.2±1.2	−130±20	−25±5
**D332G**	70.1±1.5	360±40	33±8	88.6±2.0	−145±40	−24±7
**D332P**	71.6±2.0	360±25	33±7	89.9±2.0	−160±30	−25±5
**E334L**	67.4±1.3	300±10 (1–3)	25±4	96.0±1.5	−145±20	−24±3
**E334Q**	70.1±1.5	370±25	32±6	89.0±1.5	−145±20	−41±10

**N ↔ I ↔ 1/3 I_3_ ↔ U**										
	**T_N-I_ (°C)**	**ΔH_N-I_(T_N-I_) (kJ·mol^−1^)**	**ΔG_N-I_(298) (kJ·mol^−1^)**	**T_I-U_ (°C)**	**ΔH_I-U_(T_I-U_) (kJ·mol^−1^)**	**ΔG_I-U_(298) (kJ·mol^−1^)**	**T_I-In_ (°C)**	**ΔH_I-In_(T_I-In_) (kJ·mol^−1^)**	**ΔG_I-In_(343) (kJ·mol^−1^)**	**ΔG_N-U_(298) (kJ·mol^−1^)**
**Δ10ct-PDZ3** b	51.6±3.0	250±20	28±8	78.5±3.0	215±10	17±6	124.6±4	−135±15	−27±5	28±10
**E401R**	64.4±3.5	315±30	57±10	87.5±7.0	300±25	35±12	127.7±4	−115±20	−24±6	51±20

*The error intervals were calculated as described in the text. The values of thermodynamic magnitudes for the U⇆In and I⇆In equilibriums were estimated for a P_ref_ = 100 µM. The **heat capacity functions** obtained from the fittings, in kJ·K^−1^·mol^−1^ were: **PDZ3:** C_pN_ = −9.23+(0.095*T); C_pU_ = 2.91+(0.064*T); C_pIn_ = −99.0+(0.347*T); **D332G:** C_pN_ = −9.02+(0.093*T); C_pU_ = 11.42+(0.045*T); C_pIn_ = −113.0+(0.389*T); **D332P:** C_pN_ = −12.8+(0.103*T); C_pU_ = 6.69+(0.058*T); C_pIn_ = −105.6+(0.364*T); **E334L:** C_pN_ = −24.4+(0.143*T); C_pU_ = −10.7+(0.104*T); C_pIn_ = −38.4+(0.170*T); **E334Q:** C_pN_ = −7.5+(0.085*T); C_pU_ = 16.4+(0.029*T); C_pIn_ = −85.5+(0.309*T); **Δ10ct-PDZ3:** C_pN_ = −9.7+(0.096*T); C_pU_ = 19.1+(0.117*T); C_pIn_ = −30.4+(0.131*T); C_pI_ = −31.9+(0.146*T); **E401R:** C_pN_ = −13.6+(0.107*T); C_pU_ = −34.4+(0.144*T); C_pIn_ = −79.1+(0.284*T); C_pI_ = −34.6+(0.138*T).

a Data taken from [21].

b Data taken from [24].

### FTIR Secondary Structure Analysis of PDZ3-mutants Native State

**Figure 5:**
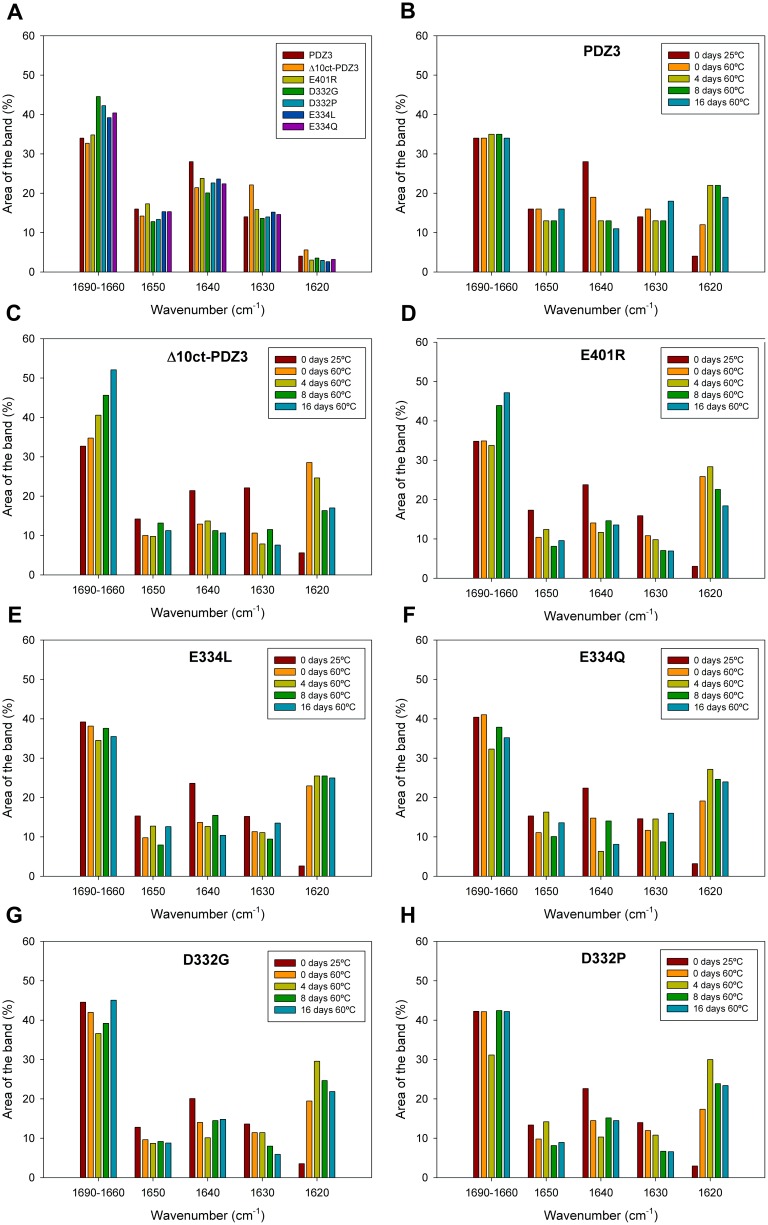
FTIR structural analysis of PSD95-PDZ3 mutants. Bars representation of the different components of the deconvoluted FTIR spectra acquired in 50(A) at 25°C for PDZ3, Δ10ct-PDZ3, and single-point mutants. Upon incubation at 60°C for several time-periods for PDZ3 (B), Δ10ct-PDZ3 (C), E401R (D), D332G (E), D332P (F), E334L (G), E334Q (H). The results for PDZ3 have been published previously [22] and are shown here for comparative purposes.

Band deconvolution of the amide I’ spectra of PDZ3 previously acquired in native conditions (25°C, 50 mM potassium phosphate pH 7.5) generated six main-bands, centered at 1680, 1670, 1660, 1650, 1640 and 1630 cm^−1^, as well as three-minor bands at 1692, 1620 and 1606 cm^−1^ [22]. Since the same components have been reported for Δ10ct-PDZ3, we concluded that Δ10ct-PDZ3 keeps a typical PDZ fold [24]. Nevertheless, some rearrangement exists. Strikingly, deletion of the α3 helix implies only 2% decrease on the α-helix band (1650 cm^−1^) (Table S1). Besides FTIR is a low-resolution technique, one plausible explanation could be that the α3 helix packs against the flexible β-sheet (strands β2, β3, β4) which, in turn, packs against the short α1 helix. Thus, the deletion could result on a better accommodation of the flexible β-sheet and on a stronger packing of α1 helix. In fact, the flexible β-sheet component, centered at 1640 cm^−1^, diminished for Δ10ct-PDZ3 with respect to PDZ3, whereas the component for the stable β-sheet (attributed to the β1 and β5 strands in PDZ3), centered at 1630 cm^−1^, was clearly increased (22% for Δ10ct-PDZ3 *versus* 14% for WT) (Figure 5 and Table S1). This concurs with an increased packing of the β-sheet arranged by β2, β3 and β4 strands surely by improving the hydrogen bonding network. Therefore, this increased packing of the β-sheets might well be the reason that, despite the decrease of the cooperative unit after truncation of α3 helix (roughly a 10%), the stability of Δ10ct-PDZ3 does not drop dramatically with respect to PDZ3 [24] (Table 2).

We have also measured FTIR spectra at 25°C for all PDZ3 mutants (Figure 5, panel A). Irregular secondary structures (1690–1660 cm^−1^) clearly increase in mutants D332P/G and E334Q/L located at the β2–β3 loop, but not in E401R which is present at the α3 helix. This would imply some unpacking resulting from destabilization of the native state upon mutation of residues Asp332 and Glu334, which in fact is detected by DSC experiments (Table 2). In contrast, E401R and Δ10ct-PDZ3 spectra do not show differences in this region when compared to PDZ3.

Glu401 is located at the C-terminus of the α3 helix; therefore, the introduction of a positive charge upon E401R mutation should allow for an interaction with the last carboxyl of the helix, decreasing the electric dipole of the α-helix and, as a consequence, stabilizing it. On the other hand, an opposing effect arises, because the mutation has precluded a salt-bridge with the β2–β3 loop. Thus, the α-helix band (1650 cm^−1^) remains essentially the same when compared to PDZ3 (17% and 16% respectively; Table S1). Concerning bands for the flexible β-sheet (identified to come from strands β2, β3, β4 in PDZ3 [22]) and the stable and long β-sheet (from strands β1 and β5) the change is, although less obvious, in the same sense to the previously observed for Δ10ct-PDZ3 [24], *i.e.*, the band at 1630 cm^−1^ increases at the expense of the band at 1640 cm^−1^ (Figure 5, panel A). Thus, an improvement of the hydrogen bonding network of the flexible β-sheet occurs upon removing or diminishing the packing of the extra-helix α3. This implies a reorganization of the native β-sheets in the native state of these mutants, which might well be related to the monomeric species detected by DSC for the intermediate states of Δ10ct-PDZ3 and E401R. In the rest of PDZ3 constructs and mutants such a monomeric stoichiometry has not been detected.

Mutation of residue Glu334 causes a higher content of irregular secondary structures (1690–1660 cm^−1^; 39% and 40% for E334L and E334Q, respectively) than that in PDZ3 (34%; panel A in Figure 5 and Table S1). The α-helix component remains the same. The band for the flexible β-sheet has decreased a bit in both mutants (24% and 22% for E334L and E334Q, respectively, *versus* 28% for PDZ3), whereas the band for the stable and long β-sheet (strands β1 and β5) remains more or less the same. Thus, mutations altering the native interaction of Glu334 with the α3 helix subsequently unpack the β2–β3 loop.

Definitely, with respect to mutations involved in the contacts between the α3 helix and the domain, it appears that bands for irregular secondary structures (1690–1660 cm^−1^) are the ones that have been increased at the cost of the flexible β-sheet (1640 cm^−1^) when the interaction Glu334-Arg399 (between β2–β3 loop and α3 helix ) is lost, whereas there exists a improvement of the β-sheet hydrogen bonding network in the case of disrupting the interaction Lys355-Glu401 (contacting strand β4 and α3 helix), which is similar, although less obvious, to the achieved by truncation of helix α3.

Finally, mutation of residue Asp332 causes the highest content of irregular secondary structures (1690–1660 cm^−1^) among mutants studied in this work, being 45% and 42% for D332G and D332P respectively, *versus* 34% for PDZ3 (panel A in Figure 5 and Table S1). This concurs after mutation of a residue located in the exposed-to-solvent β2–β3 loop, specially increasing flexibility when the introduced residue is a Gly. The succinimide ring formed by this residue in PDZ3, that may drive to a decreased flexibility of the β2–β3 loop [20], cannot be spontaneously formed in these mutants, but is mimicked by the Pro mutation. A scarce reduction of the α-helix component has occurred upon mutation of residue Asp332 (13% for both D332G and D332P *versus* 16% for PDZ3) (Table S1). This could be due to a decrease of the α3 helix content. The band for the flexible β-sheet has slightly decreased in both, 20% and 23% for D332G and D332P, respectively, *versus* 28% for PDZ3 (panel A in Figure 5 and Table S1). However, the stable and long β-sheet (strands β1 and β5) remains the same. This is because in these cases, bands for irregular secondary structures (1690–1660 cm^−1^) are the ones that have increased.

In summary, the increase in irregular structures achieved by Asp332 might be a consequence of the probable contribution of such residue to the interactions between the α3 helix and the β2–β3 loop, in the same way than the near residue Glu334, both pointing towards Arg399 of α3 helix in the X-ray structure (Figure 1). Thus, the disruption of the interactions between these two PDZ3 regions will drive to an additional destabilization of the prone-to-aggregation motif of PDZ3, comprised by strands β2 to β4 [22], which in fact decreases the native-state stability as seen by DSC experiments (Table 2).

### Misfolding and Aggregation of Δ10ct-PDZ3 and Point Mutations

**Figure 6:**
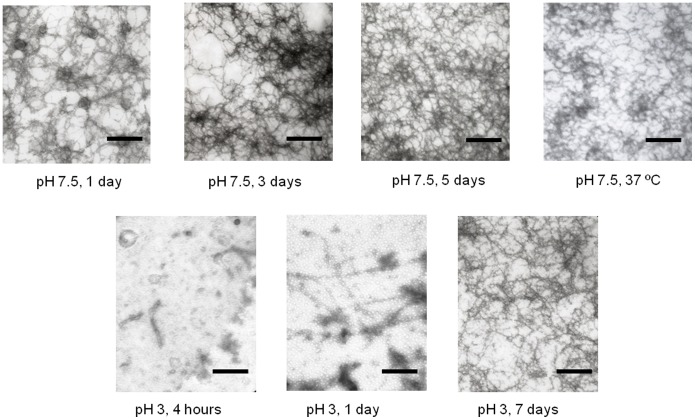
TEM analysis of PDZ3-mutants supramacromolecular structures developed after different incubation periods. Upper row of pictures: A 727 µM solution of Δ10ct-PDZ3 in 50 mM potassium phosphate pH 7.5 was incubated for different time intervals, from left to right, 1 day, 3 days and 5 days; whereas the latter picture was taken from a sample incubated at 37°C during 1 month. The second-row panels show a TEM analysis of the supramacromolecular structures developed by Δ10ct-PDZ3 solutions at 727 µM in 50 mM glycine/HCl buffer pH 3.0 after different incubation periods. The horizontal black bar corresponds with a length of 200 nm.

To definitely confirm whether the intermediate state of Δ10ct-PDZ3, the most extreme change in the PDZ3 sequence analyzed in this work, may evolve to supramacromolecular structures, as it happens in the PDZ3 case, we incubated a 727 µM sample at 60°C for a long-time period. We followed such an evolution by DLS, which showed an increase of the hydrodynamic radius with the incubation time (Figure S1). In agreement, size exclusion chromatography of these incubated Δ10ct-PDZ3 samples at 60°C in a Superdex-75 column (GE healthcare) showed a single elution peak at the exclusion volume of the column (≥70 kDa); this was observed even with non-incubated samples when heated at 60°C just inside the column (data not shown). TEM analysis showed the appearance of fibrils after 1-day incubation. Even at 37°C, sample incubation drove to fibrils after a long-time incubation, around 1 month (Figure 6).

These results bear out noticeable differences with respect to PDZ3 and point mutations, where a variety of oligomeric species that roughly correlate with the DLS experiments were observed by chromatography [21]. Particles size increased in a more moderated way than in Δ10ct-PDZ3, having only curly fibrils after 1 month incubation at 37°C. Accordingly, TEM analysis only revealed roughly similar structures to the shown in Figure 6 at a time not shorter than one week [21]. Another interesting feature revealed from TEM and DLS analyses is the irreversible character of Δ10ct-PDZ3 fibrils, whereas in the case of PDZ3 and point mutations we observe a partial reversibility of these arrangements, which could return to the N-state after cooling down to 20°C and/or dilution of the PDZ3 solution [21], [22].

Therefore, we can conclude that the α3 helix seems to protect to some extent the PDZ domain from misfolding, since the precursory intermediate state is clearly lowly populated being the following misfolding route kinetically slow and partially reversible when α3 helix is attached to the domain.

To understand the fibrils organization mechanism in Δ10ct-PDZ3 we measured fluorescence emission by ThT (thioflavine T) and ANS (8-aniline sulphonic acid). Under the same buffer conditions used in the previous experiments, we prepared samples at 40 and 727 µM protein concentration, including saturating concentrations of ThT (12.5 µM) or ANS (20 µM). The progressive heating of these samples from 20 to 60°C revealed a slight increase of fluorescence at 40°C for the higher protein concentration and above 50°C for the lower one (Figure S2, left panels), in full agreement with the populations analysis formerly done for Δ10ct-PDZ3 [24]. Such increase is due to the binding of both chemicals to newly solvent-exposed hydrophobic pockets in the domain (ANS) and the simultaneous appearing of β-aggregates (ThT). We also observed a parallel increase in the fluorescence intensity with protein concentration.

A further incubation of these samples at 60°C for several hours (Figure S2, right panels) showed that fluorescence emission dramatically increased at the very beginning of the incubation, which reveals the lack of a lag-phase for the association of monomers in the I_n_ state, as it happens in PDZ3 and point mutations. Nevertheless, the maximum intensity is reached at roughly 30 min in the case of ThT and 350 min for ANS, whereas in the PDZ3 analysis these maximums where found at 100 min and 400 min respectively [21]. The following drop of fluorescence is due to the increase in viscosity of the solution, which generates an increase of light dispersion phenomena as a consequence of the appearance of the poorly soluble supramacromolecular structures [25], [26], [27]. When the incubation was done with a protein concentration of 40 µM the maximums are difficult to be evaluated since the curves do not drop appreciably, because aggregates are in a clearly lower amount than at 727 µM. In all cases, fibrillation kinetics in the presence of both ThT and ANS (Figure S2, right panels) lack the lag phase featuring amyloid fibril formation and behave as it has been described for curved fibrils, i.e. those worm-like fibrils formed by β2-microglobulin [28].

The population distribution of the different Δ10ct-PDZ3 species (Figure 3) also shows that the folding intermediate destabilizes when pH drops, as in the PDZ3 case, since its relative population decreases as a whole from 100% at pH 4.0 to no more than 20% at pH 2.5. To understand how this decrease may affect the misfolding route, we have incubated at 60°C (where the intermediate species are maximally populated) various Δ10ct-PDZ3 samples at 727 µM and pH 3.0. Inspection by TEM (Figure 6) showed that after 4 hours incubation proto-fibrils were present, becoming longer after 1 day; however, it is necessary to wait until 1 week to observe abundant fibrils, which were straighter and longer than the ones obtained at pH 7.5 (Figure 6). We also performed ThT and ANS fluorescence emission measurements at pH 3.0 with these protein samples (Figure S3). ANS fluorescence did not show any lag-phase but, opposite to what seen at pH 7.5, ThT fluorescence did. Thus, in full agreement with TEM, we have some intermediates populating from the very beginning but nucleation is necessary prior to the elongation phase that gives rise to straight and long protofibrils.

Finally, we measured the fluorescence of all these samples after cooling down to 25°C with the goal of checking the irreversibility of Δ10ct-PDZ3 fibrils. In agreement with our previous evidence, fluorescence emission remained almost unaltered, even after three days at room temperature (Figure S4), which confirms the irreversible nature of Δ10ct-PDZ3 fibrils. In the case of point mutations, reversibility was similar to the previously reported for PDZ3 [21].

### Structural Analysis of PDZ3-mutants Intermediate State and Misfolding

The conformational changes that undergo PDZ3 upon incubation at 60°C have been already published [22]. In short (Figure 5, panel B), the component for the flexible β-sheet dramatically decreased while concomitantly the component for β-aggregates increased. The more structured part of the native anti-parallel β-sheet and the α-helix components also scarcely decreased, although the differences were too small to be unequivocally determined.

The conformational changes in Δ10ct-PDZ3 upon incubation at 60°C have been studied here, after 5 min of equilibration and after incubation for 4, 8, and 16 days (Figure 5, panel C). After 5 min at 60°C Δ10ct-PDZ3 showed an increase in the region corresponding to loops/turns, and this increase became progressively greater upon incubation; *i.e.*, at 25°C these bands comprised 33% of the spectrum area whereas after 16 days the value reached 52% (Table S1). Although some decrease of the α-helix component occurred just upon 5 min incubation, the value increased after 8 days but did not recover the initial value (Figure 5, panel C). For PDZ3, 4 days of incubation were necessary to detect such a decrease and the recovery after 16 days of incubation was completed (Figure 5, panel B). Also, just by incubating for 5 min the component for the flexible β-sheet dropped in Δ10ct-PDZ3 and stayed more or less the same until 16 days (Figure 5, panel C). This was also the case for PDZ3 but, again, the drop occurred after 4 days (Figure 5, panel B). In contrast to PDZ3, which maintained the band for the stable β-sheet more or less similar during 16 days, in Δ10ct-PDZ3 this band clearly decreased by just incubating for 5 min. This means that both native β-sheets, the flexible and the stable ones, are lost in favour of the β-aggregate component in Δ10ct-PDZ3. However, the β-aggregate component is maximal at day 4, reaching 29% of the area of the spectrum, decreasing thereafter in favour of the above mentioned increase of the loop/turn component. After 16 days, the β-aggregate component comprises 17% of the spectra (Table S1).

Therefore, in the case of PDZ3, the α3 helix diminishes to some extent the aggregation tendency by maintaining the integrity of the native β-sheet formed by chains β1 and β5. In the case of Δ10ct-PDZ3, apart from β5 chain becoming the C-terminal of the molecule, there is a reorganization of the native β-sheet which propitiates chains β1 and β5 to become precursors of aggregation together with chains β2 to β4. Although the aggregation process is faster in the Δ10ct-PDZ3 variant, the β-aggregate component comprises the same percentage of the spectra after 16 days incubation as in the case of the complete PDZ3 (17% *versus* 19%). These differences would be indicative of the presence of the monomeric intermediate state in Δ10ct-PDZ3, which immediately appears upon heating at 60°C. However, at the end the trimeric intermediate aggregates more or less in the same way for PDZ3 than for Δ10ct-PDZ3.

The conformational changes that undergo the set of PDZ3 mutants upon incubation at 60°C have been also studied by FTIR after 5 min of equilibration, as well as after incubation for 4, 8, and 16 days at this temperature (Figure 5). As in the case for Δ10ct-PDZ3, in the E401R mutation the aggregation band (1620 cm^−1^) immediately appeared upon heating at 60°C and further decreased (Figure 5, panel D), this decrease being also concomitant with an increase in the region corresponding to loops/turns. However, mutations involving the β2–β3 loop behaved more or less as PDZ3 (Figure 5, panels E-H), that is, progressively increased the area of the aggregation band up to four days of incubation and maintaining or so its value at the end. Concerning to the main secondary structures reorganizing to generate the aggregation band (1620 cm^−1^) both the flexible (1640 cm^−1^) and the stable β-sheet (1630 cm^−1^) are involved in all of the mutants, but specially in those in α3 helix.

In summary, albeit some differences, the main change for all the spectra is the earlier and dramatic increase of the 1620 cm^−1^ band (corresponding to an ordered β-aggregate), mainly for Δ10ct-PDZ3 and E401R (up to 29% and 26%, respectively, *versus* 12% for PDZ3), achieved just after 5 min of incubation. In concordance, this increase should be related with the conformational reorganization of the whole native β-sheet that should drive to the monomeric intermediate. In general, mutants located at the α3 helix (Δ10ct-PDZ3 and E401R) are more prone to aggregation than PDZ3, in full agreement with TEM and DLS evidence, although after 16 days the 1620 cm^−1^ component is the same than that for PDZ3 (∼18%), probably because the predominance of the trimeric intermediate. Nevertheless, mutants D332G/P and E334L/Q, both into the prone-to-aggregation region of PDZ3, show a greater aggregation tendency than PDZ3 just upon initial incubation and maintain the β-aggregate band at around 25%. This concurs with the absence of the monomeric state in the DSC traces fitting. In consequence, it can be claimed that although all these mutations generate proteins more prone to aggregation than the wild-type PDZ3, those directly affecting the α3 helix stabilize the monomeric intermediate whereas those affecting the β2–β3 loop stabilize the trimeric intermediate.

## Discussion

**Figure 7:**
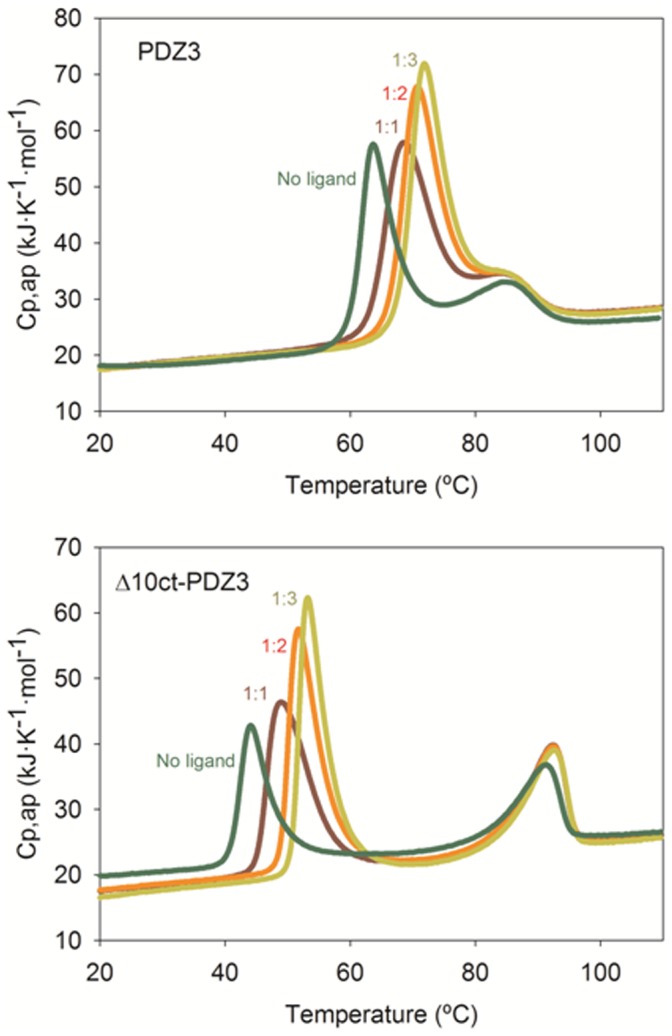
DSC thermal unfolding profiles for wild-type PDZ3 and Δ10ct-PDZ3 obtained at different molar proportions of a high affinity ligand, KKETAV. Concretely, 1∶0 (no ligand), 1∶1, 1∶2 and 1∶3 from left to right. Protein concentrations were 1.6 and 1.4 mg·mL^−1^ respectively in potassium phosphate buffer pH 7.5.

The comprehensive interpretation of the folding/misfolding results of Δ10ct-PDZ3 and PDZ3 point mutations, using as a reference the whole PDZ3 construction including the α3 helix (residues 302 to 402 of PSD-95 protein), drives us to conclude that such C-terminal helix is a regulatory element of the folding of this PDZ domain. Thus, truncation from PDZ3 not only favours the population of an associated intermediate previously detected in PDZ3, but also drives to a higher tendency to generate supramacromolecular assemblies. Although the DSC analysis predicts a trimeric stoichiometry for the early association stages as it happened in PDZ3 [21], in the case of Δ10ct-PDZ3 we could not detect such trimers by size-exclusion chromatography or DLS, where higher-size particles were observed. The fast inter-association of trimeric particles might preclude their detection, as it was previously reported for other examples [29]. TEM and FTIR analyses confirm such a faster misfolding, in full agreement with ThT and ANS kinetics too. Moreover, aggregates are irreversible, whereas the presence of the α3 helix at the C-terminus generated some degree of reversibility in PDZ3 fibrils organization [21]. Despite the obvious improving of fibrillation kinetics, which becomes faster, the longest-time incubated samples showed a similar percentage of β-aggregates which suggest that misfolding thermodynamics are not significantly different, in full agreement with DSC results (Table 2). Let remark that the intermediate and/or the misfolded species described do not appear to bind the high-affinity ligand KKETAV, as demonstrate some DSC experiments with PDZ3 and Δ10ct-PDZ3 carried out at different concentrations of KKETAV. As shown in Figure 7 the peptide is only recognized by the native state, which is shown by the shift to higher temperatures of the first endotherm (reporting for the native to intermediate state equilibrium) with ligand concentration, whereas the second transition (reporting for the intermediate to unfolded state equilibrium) is not influenced by the presence of ligand.

To get insight into the molecular origins of the misfolded species we can take a look to Figure 1 where it is observed that the α3 helix packs against the PDZ3 fold, being possible the interaction with some charged residues of the β2 to β4 region. Therefore, the absence of helix α3 will increase solvent accessibility of such a region, which may drive to a higher tendency to partial unfolding and self-association. On the other hand, the native state Δ10ct-PDZ3 contains a stronger hydrogen bonding network than that in PDZ3, as seen by FTIR. Similar measurements fully confirm a faster aggregation in the case of Δ10ct-PDZ3 than in PDZ3, and also show that the origins of the misfolded species would reside in the two native β-sheets, the flexible and the stable ones (Figure 1), which are lost in favour of the β-aggregate component upon misfolding. This is opposite to what happens with PDZ3, were the main precursor for the β-aggregate component is the flexible β-sheet, thus indicating that the rearrangement of this flexible element on the stable β-sheet upon removing α3 helix makes the later more prone to aggregation. This feature was already predicted by Tango algorithm which revealed residues 335–344 (region β3) and 384–392 (region β5) as the most prone to aggregation [21], [22]. Thus, the β5 region would produce a worse packing when the extra-helix is absent, since *i)* it is at the C-terminus, and *ii)* the stable β-sheet has changed its hydrogen bonding pattern. Consequently, in this situation both β-sheets can drive to the organization of supramacromolecular structures. Nevertheless, in the case of the whole PDZ3 domain some protection from aggregation is made by the α3 helix, well enough to preserve the integrity of the most stable β-sheet organized between strands β1 and β5 in the precursory intermediate and to slow down misfolding, causing also some degree of reversibility in such a process [22]. These differences are also reflected in TEM images, showing that the monomeric intermediate drives to protofibrils, whereas worm-like fibrils come from the trimeric species previously described for PDZ3 [21]. Therefore, this evidence points to different competing misfolding pathways for PDZ3 directly related to the extra-α3 helix influence.

The experimental evidence of a drop in population of the intermediate species when pH drops at values below 3, gives some light about the possible regulatory mechanism of PDZ3 folding/misfolding by the α3 helix. Thus, Glu and Asp residues appear as the responsible of such a destabilization. In this way, we have previously described that this extra-helical element plays a regulatory role upon ligand binding to PDZ3, being mainly due to the interaction of Arg399 (α3 helix) with Glu334 (β2–β3 loop). As we mentioned, there exist another well established salt-bridge between residues Glu401 (α3 helix) and Lys355 (β4 strand), but this one does not seem to contribute appreciably to the binding process [18]. Nevertheless, our folding studies reveal that the opposite is true, having qualitatively similar effects in PDZ3 by mutating Glu401 and by truncating α3 helix. Thus, both variants display a roughly similar four-state unfolding model, as well as FTIR structural features quite similar upon misfolding, where the bands for both native β-sheets, the flexible and the stable, progressively diminished during incubation concomitantly with the increase of the β-aggregate band. Finally, the aggregation band diminished in favour of an increase of the loops/turns component. This behaviour is different to PDZ3 and the rest of mutants, E334L/Q and D332P/G, where the decrease of the stable β-sheet is not so evident and the region corresponding to loops/turns progressively decreases during incubation, whereas the α-helix band fluctuates around the initial value.

Thus, from the study of mutations E334Q/L and E401R, checking the contacts between the α3 helix and the domain, it appears that irregular secondary structures increase at the cost of the flexible β-sheet when the interaction Glu334-Arg399 (between β2–β3 loop and helix α3) is disrupted, whereas there exists a net improvement of the β-sheet hydrogen bonding network in the case of altering interaction Lys355-Glu401 (contacting α3 helix and β4 strand) and both β-sheets contribute to the arrangement of misfolding aggregates. These features support the conclusion that a different intermediate appears when the α3 helix is not involved in misfolding.

On the other hand, the highest increase of irregular structures achieved by Asp332P/G mutants might be a consequence of the probable contribution of Asp332 to the interactions between the α3 helix and the β2–β3 loop, in the same way than the near residue Glu334, both pointing towards Arg399 of α3 helix in the X-ray structure [20]. Thus, the disruption of the interactions between these two PDZ3 regions will drive to an additional destabilization of the prone-to-aggregation region of PDZ3, comprised by strands β2 to β4 [22]. This result suggests that, equal to binding, succinimide-ring formation at Asp332 develops a negative impact on folding, since is able to accelerate PDZ3 misfolding. This is not a surprising result, a parallel effect has been described upon succinilation of Asn residues in γ-S crystalline, which generates the opaque deposits found in ocular cataracts as a consequence of the decrease of solubility of this succinilated protein [30]. Moreover, it has been postulated elsewhere that spontaneous Asp to succinimide to iso-Asp transformations are associated to a higher β-aggregation tendency in some peptides [31], including the Alzheimer’s Aβ peptide [32].

Definitely, our results strongly suggest that α3 helix can be a natural regulator of the misfolding kinetics of PDZ3. Probably, there exist evolutionary reasons for this functionality since protein evolution favours the suppression of misfolding and the assessment of well-defined and cooperative three-dimensional structures. It is known that consecutive homologous domains in large multi-domain proteins have evolved to sequence identities of less than 40% to avoid co-aggregation [33]. According to our evidence, the insertion of an extra α-helix can preclude aggregation, as well as mutational helix stabilization, as shown elsewhere [34]. To the best of our knowledge, this might well constitute the first example where an extra-element, intended to link the PDZ3 domain to the following SH3 in PSD-95 and in some other members of the MAGUK family, not only regulates the binding abilities of this domain [18], but it can also protect PDZ3 from misfolding and aggregation, by almost suppressing the aggregation of one of its β-sheets (the one organized by strands β1 and β5) and by significantly slowing down the misfolding of the another one (organized by strands β2, β3 and β4).

## Supporting Information

Figure S1 **DLS experiments carried out at pH 7.5 with Δ10ct-PDZ3.** A protein solution in potassium phosphate pH 7.5 buffer and at a concentration of 727 µM was heated initially from room temperature to 60°C, where the I_n_ species should be most populated according to DSC analysis. It was then kept at 60°C, at which point the mass evolution of the species as a function of incubation time was recorded. Vertical dashed lines represent average particle sizes of 6 nm and 30 nm respectively.
(DOCX)

Figure S2 **Fluorescence measurements of PDZ3 mutants in the presence of ThT and ANS at pH 7.5.** Left panels: temperature scanning until 60°C of fluorescence emission of PDZ3 samples in the presence of ANS or ThT in the case of Δ10ct-PDZ3, or in the presence of ThT in the case of PDZ3 mutants. Right panels: Growth kinetics followed by fluorescence emission of PDZ3 solutions described in the respective left panels. Buffer conditions were 50 mM potassium phosphate pH 7.5 in the presence of either 12.5 µM ThT or 20 µM ANS.
(DOCX)

Figure S3 **Fluorescence measurements of Δ10ct-PDZ3 in the presence of ThT and ANS at pH 3.0.** The growth kinetics followed by fluorescence emission of a 727 µM Δ10ct-PDZ3 solution in 50 mM glycine/HCl buffer pH 3.0 in the presence of either 12.5 µM ThT or 20 µM ANS.
(DOCX)

Figure S4 **Emission fluorescence spectra of ThT (upper panel) or ANS (lower panel), after cooling down to 25**°C **a Δ10ct-PDZ3 solution.** Protein concentration was 727 µM, in 50 mM potassium phosphate pH 7.5 in the presence of either 12.5 µM ThT or 20 µM ANS. These solutions were previously incubated at 60°C during the several hours to perform growth kinetics. Yellow line, after 1 min at 25°C; blue after 1 hour; brown, after 3 days; purple, the spectra of native Δ10ct-PDZ3.
(RAR)

Table S1 Band deconvolution of the amide I’ FTIR spectra of Δ10ct-PDZ3 and some PDZ mutants in native conditions (25°C, 50 mM potassium-phosphate buffer, pH 7.5) and upon incubation at 60°C for several time-periods.
(DOCX)
