# Supplementary figures and images for: The Impact of Extra-Domain Structures and Post-Translational Modifications in the Folding/Misfolding Behaviour of the Third PDZ Domain of MAGUK Neuronal Protein PSD-95
Source: PLoS One. 2014 May 20;9(5):e98124. doi: 10.1371/journal.pone.0098124 (PMC4028313; doi:10.1371/journal.pone.0098124)

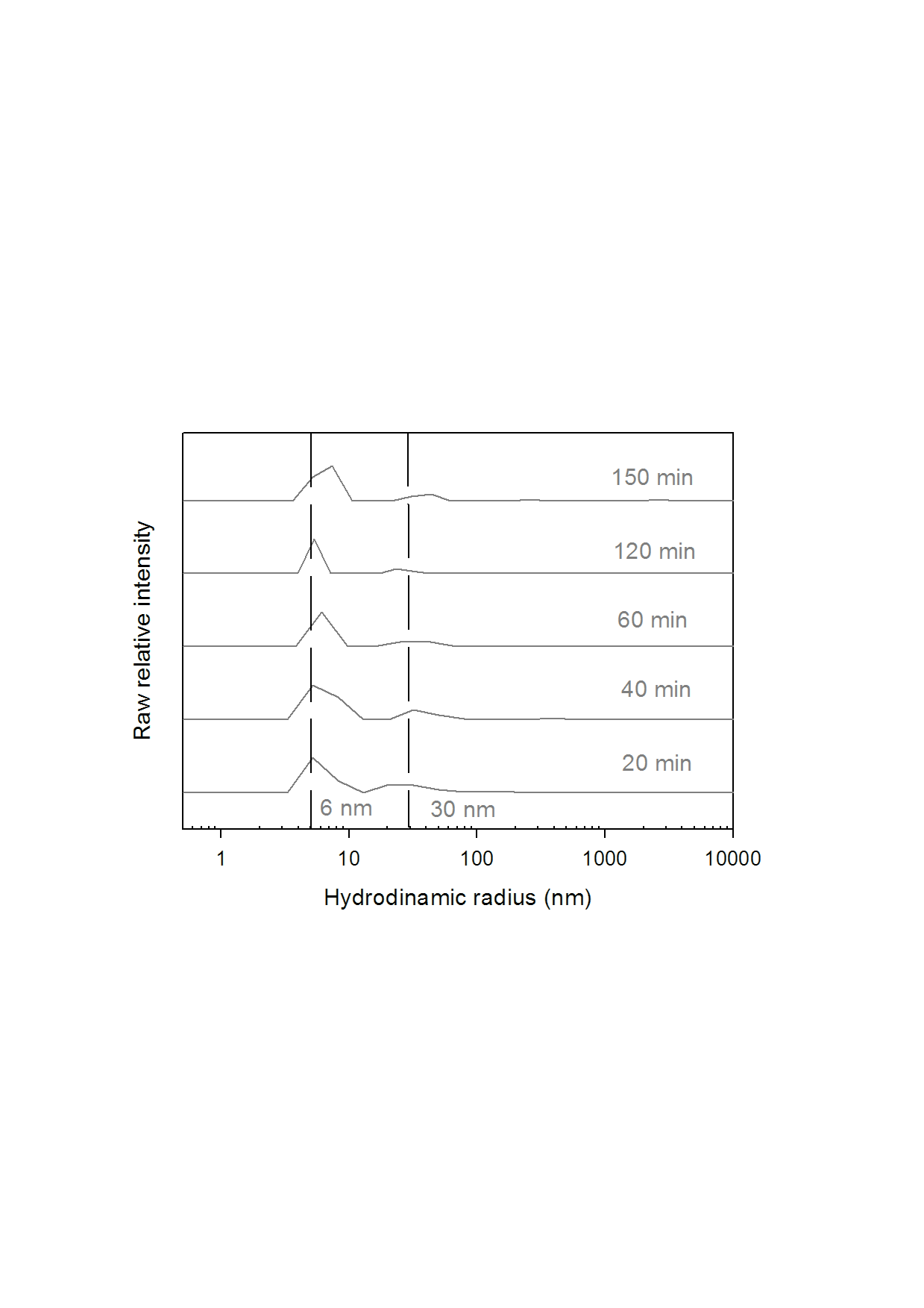

Supplement: Figure S1 — DLS experiments carried out at pH 7.5 with Δ10ct-PDZ3. A protein solution in potassium phosphate pH 7.5 buffer and at a concentration of 727 µM was heated initially from room temperature to 60°C, where the In species should be most populated according to DSC analysis. It was then kept at 60°C, at which point the mass evolution of the species as a function of incubation time was recorded. Vertical dashed lines represent average particle sizes of 6 nm and 30 nm respectively. (DOCX) [file pone.0098124.s001.docx]

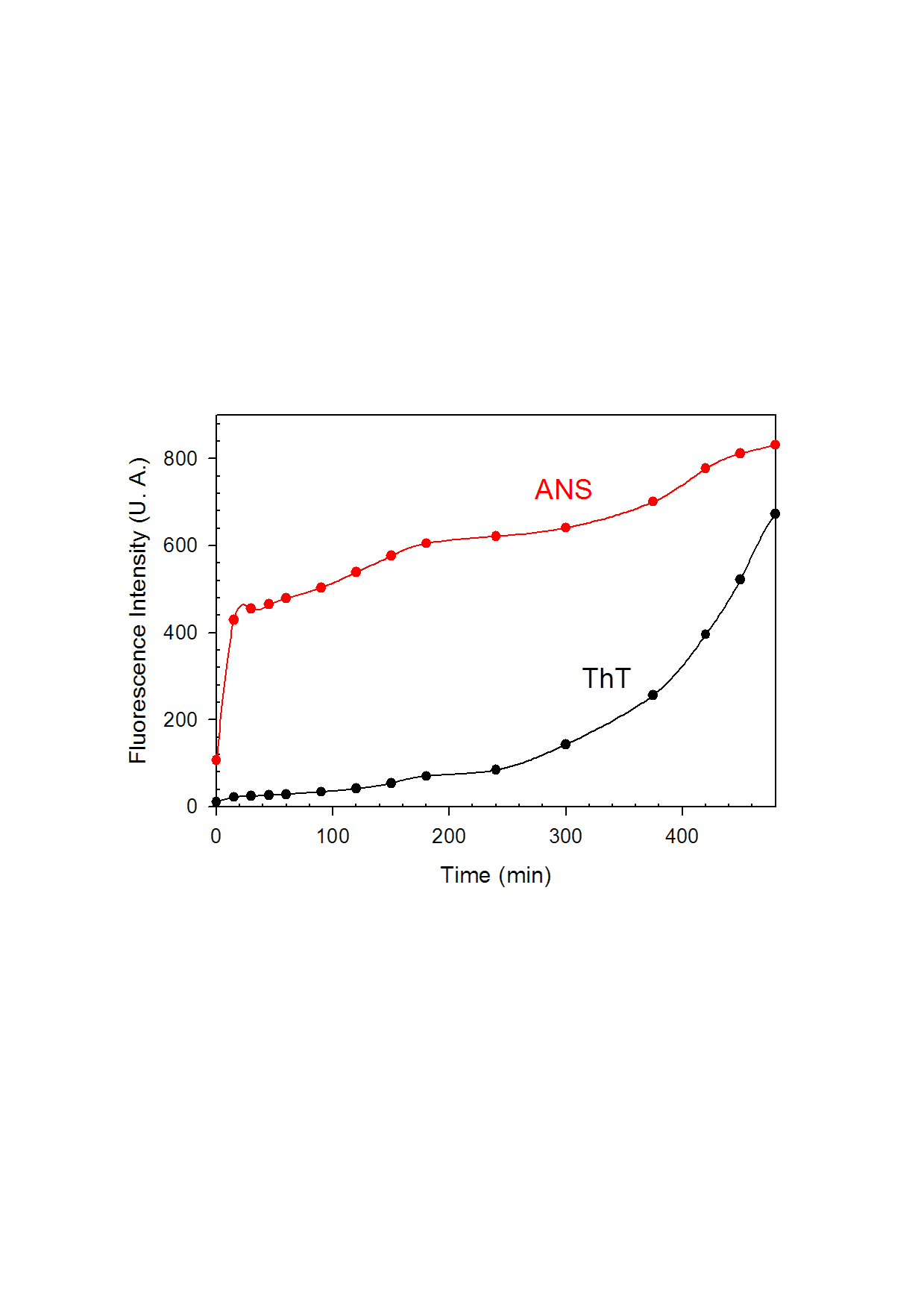

Supplement: Figure S3 — Fluorescence measurements of Δ10ct-PDZ3 in the presence of ThT and ANS at pH 3.0. The growth kinetics followed by fluorescence emission of a 727 µM Δ10ct-PDZ3 solution in 50 mM glycine/HCl buffer pH 3.0 in the presence of either 12.5 µM ThT or 20 µM ANS. (DOCX) [file pone.0098124.s003.docx]
