# Supplementary material for: The Impact of Extra-Domain Structures and Post-Translational Modifications in the Folding/Misfolding Behaviour of the Third PDZ Domain of MAGUK Neuronal Protein PSD-95
Source: PLoS One. 2014 May 20;9(5):e98124. doi: 10.1371/journal.pone.0098124 (PMC4028313; doi:10.1371/journal.pone.0098124)
Supplement: Figure S2 — Fluorescence measurements of PDZ3 mutants in the presence of ThT and ANS at pH 7.5. Left panels: temperature scanning until 60°C of fluorescence emission of PDZ3 samples in the presence of ANS or ThT in the case of Δ10ct-PDZ3, or in the presence of ThT in the case of PDZ3 mutants. Right panels: Growth kinetics followed by fluorescence emission of PDZ3 solutions described in the respective left panels. Buffer conditions were 50 mM potassium phosphate pH 7.5 in the presence of either 12.5 µM ThT or 20 µM ANS. (DOCX) [file pone.0098124.s002.docx]

**









**
