# Supplementary material for: The Impact of Extra-Domain Structures and Post-Translational Modifications in the Folding/Misfolding Behaviour of the Third PDZ Domain of MAGUK Neuronal Protein PSD-95
Source: PLoS One. 2014 May 20;9(5):e98124. doi: 10.1371/journal.pone.0098124 (PMC4028313; doi:10.1371/journal.pone.0098124)
Supplement: Table S1 — Band deconvolution of the amide I’ FTIR spectra of Δ10ct-PDZ3 and some PDZ mutants in native conditions (25°C, 50 mM potassium-phosphate buffer, pH 7.5) and upon incubation at 60°C for several time-periods. (DOCX) [file pone.0098124.s005.docx]

**Table S1.** Band deconvolution of the amide I’ FTIR spectra of Δ10ct-PDZ3 and some PDZ mutants in native conditions (25 ºC, 50 mM potassium-phosphate buffer, pH 7.5) and upon incubation at 60ºC for several time-periods.

| **25ºC, 0 days** | **PDZ3 ^a^** | | **Δ10ct-PDZ3 ^b^** | | **E401R** | | **D332G** | | **D332P** | | **E334L** | | **E334Q** | |
| --- | --- | --- | --- | --- | --- | --- | --- | --- | --- | --- | --- | --- | --- | --- |
|  | **Center**  **(cm-1 )** | **Area**  **(%)** | **Center**  **(cm-1 )** | **Area**  **(%)** | **Center**  **(cm-1 )** | **Area (%)** | **Center (cm-1 )** | **Area (%)** | **Center (cm-1 )** | **Area (%)** | **Center (cm-1 )** | **Area (%)** | **Center**  **(cm-1 )** | **Area**  **(%)** |
| β-turns | 1694 | 1 | 1692 | 2 | 1692 | 3 | 1691 | 9 | 1691 | 7 | 1691 | 5 | 1691 | 6 |
| High-frequency antiparallel β-sheet | 1680 | 8 | 1677 | 9 | 1680 | 8 | 1679 | 10 | 1679 | 10 | 1679 | 9 | 1679 | 9 |
| β-turns | 1669 | 9 | 1669 | 7 | 1669 | 8 | 1668 | 9 | 1668 | 9 | 1668 | 9 | 1668 | 9 |
| **Loops/Turns** | **1659** | **16** | **1659** | **15** | **1659** | **16** | **1659** | **16** | **1659** | **17** | **1659** | **17** | **1659** | **17** |
| α-helix | 1650 | 16 | 1649 | 14 | 1649 | 17 | 1649 | 13 | 1649 | 13 | 1649 | 15 | 1649 | 15 |
| **Flexible β-sheet/Random coil** | **1640** | **28** | **1641** | **21** | **1640** | **24** | **1641** | **20** | **1641** | **23** | **1641** | **24** | **1640** | **22** |
| Low-frequency antiparallel β-sheet | 1630 | 14 | 1631 | 22 | 1631 | 16 | 1630 | 14 | 1630 | 14 | 1630 | 15 | 1630 | 15 |
| **WL/Amyloid** | **1620** | **4** | **1621** | **6** | **1621** | **3** | **1621** | **4** | **1621** | **3** | **1621** | **3** | **1621** | **3** |
| Side-chains | 1606 | 4 | 1607 | 4 | 1605 | 5 | 1605 | 5 | 1605 | 4 | 1605 | 4 | 1605 | 4 |
| **60ºC, 0 days (5 min)** | **PDZ3 ^a^** | | **Δ10ct-PDZ3** | | **E401R** | | **D332G** | | **D332P** | | **E334L** | | **E334Q** | |
|  | **Center (cm-1 )** | **Area (%)** | **Center (cm-1 )** | **Area (%)** | **Center (cm-1 )** | **Area (%)** | **Center (cm-1 )** | **Area (%)** | **Center (cm-1 )** | **Area (%)** | **Center (cm-1 )** | **Area (%)** | **Center (cm-1 )** | **Area**  **(%)** |
| β-turns | 1695 | 1 | 1691 | 4 | 1691 | 4 | 1691 | 8 | 1691 | 8 | 1691 | 6 | 1691 | 7 |
| High-frequency antiparallel β-sheet | 1684 | 2 |  |  |  |  |  |  |  |  |  |  |  |  |
|  | 1676 | 9 | 1679 | 10 | 1679 | 10 | 1678 | 11 | 1678 | 11 | 1679 | 10 | 1678 | 10 |
| β-turns | 1669 | 7 | 1668 | 8 | 1669 | 8 | 1668 | 9 | 1668 | 9 | 1668 | 9 | 1668 | 9 |
| **Loops/Turns** | **1659** | **15** | **1659** | **13** | **1659** | **13** | **1659** | **14** | **1659** | **14** | **1659** | **13** | **1659** | **14** |
| α-helix | 1649 | 16 | 1649 | 10 | 1649 | 10 | 1648 | 10 | 1648 | 10 | 1649 | 10 | 1649 | 11 |
| **Flexible β-sheet/Random coil** | **1640** | **19** | **1641** | **13** | **1641** | **14** | **1641** | **14** | **1641** | **14** | **1641** | **14** | **1641** | **15** |
| Low-frequency antiparallel β-sheet | 1631 | 16 | 1631 | 11 | 1631 | 11 | 1630 | 11 | 1630 | 12 | 1630 | 11 | 1630 | 12 |
| **WL/Amyloid** | **1620** | **12** | **1619** | **29** | **1619** | **26** | **1620** | **19** | **1620** | **17** | **1619** | **23** | **1620** | **19** |
| Side-chains | 1606 | 3 | 1606 | 3 | 1606 | 4 | 1608 | 4 | 1609 | 4 | 1608 | 4 | 1608 | 2 |
| **60ºC, 4 days** | **PDZ3 ^a^** | | **Δ10ct-PDZ3** | | **E401R** | | **D332G** | | **D332P** | | **E334L** | | **E334Q** | |
|  | **Center (cm-1 )** | **Area (%)** | **Center (cm-1 )** | **Area (%)** | **Center (cm-1 )** | **Area (%)** | **Center (cm-1 )** | **Area (%)** | **Center (cm-1 )** | **Area (%)** | **Center (cm-1 )** | **Area (%)** | **Center (cm-1 )** | **Area**  **(%)** |
| β-turns | 1692 | 2 | 1694 | 2 | 1694 | 1 | 1695 | 1 | 1695 | 1 | 1695 | 1 | 1695 | 1 |
| High-frequency antiparallel β-sheet | 1679 | 12 | 1683 | 12 | 1681 | 11 | 1683 | 6 | 1683 | 7 | 1682 | 11 | 1683 | 5 |
| β-turns | 1668 | 8 | 1670 | 17 | 1668 | 12 | 1672 | 12 | 1669 | 15 | 1668 | 12 | 1671 | 13 |
| **Loops/Turns** | **1658** | **13** | **1659** | **10** | **1658** | **10** | **1658** | **18** | **1659** | **8** | **1659** | **10** | **1659** | **12** |
| α-helix | 1648 | 13 | 1650 | 10 | 1649 | 12 | 1647 | 9 | 1649 | 14 | 1649 | 13 | 1648 | 16 |
| **Flexible β-sheet/Random coil** | **1640** | **13** | **1640** | **14** | **1640** | **12** | **1640** | **10** | **1640** | **10** | **1640** | **13** | **1640** | **6** |
| Low-frequency antiparallel β-sheet | 1631 | 13 | 1631 | 8 | 1631 | 10 | 1631 | 11 | 1631 | 11 | 1630 | 11 | 1632 | 15 |
| **WL/Amyloid** | **1620** | **22** | **1618** | **25** | **1619** | **28** | **1619** | **30** | **1619** | **30** | **1619** | **25** | **1619** | **27** |
| Side-chains | 1606 | 4 | 1606 | 3 | 1606 | 4 | 1606 | 4 | 1606 | 4 | 1606 | 4 | 1606 | 3 |

| **60ºC, 8 days** | **PDZ3 ^a^** | | **Δ10ct-PDZ3** | | **E401R** | | **D332G** | | **D332P** | | **E334L** | | **E334Q** | |
| --- | --- | --- | --- | --- | --- | --- | --- | --- | --- | --- | --- | --- | --- | --- |
|  | **Center (cm-1 )** | **Area (%)** | **Center (cm-1 )** | **Area (%)** | **Center (cm-1 )** | **Area (%)** | **Center (cm-1 )** | **Area (%)** | **Center (cm-1 )** | **Area (%)** | **Center (cm-1 )** | **Area (%)** | **Center (cm-1 )** | **Area**  **(%)** |
| β-turns | 1692 | 2 | 1695 | 3 | 1694 | 3 | 1694 | 2 | 1694 | 2 | 1695 | 1 | 1694 | 2 |
| High-frequency antiparallel β-sheet |  |  | 1684 | 13 | 1683 | 12 | 1683 | 10 | 1683 | 11 | 1682 | 13 | 1683 | 10 |
|  | 1679 | 12 | 1676 | 7 |  |  |  |  |  |  |  |  |  |  |
| β-turns | 1668 | 8 | 1667 | 15 | 1670 | 19 | 1669 | 19 | 1670 | 20 | 1670 | 9 | 1669 | 16 |
| **Loops/Turns** | **1658** | **13** | **1659** | **7** | **1659** | **10** | **1659** | **9** | **1659** | **9** | **1659** | **15** | **1659** | **10** |
| α-helix | 1648 | 13 | 1651 | 13 | 1650 | 8 | 1650 | 9 | 1650 | 8 | 1649 | 8 | 1650 | 10 |
| **Flexible β-sheet/Random coil** | **1640** | **13** | **1641** | **11** | **1641** | **15** | **1641** | **14** | **1641** | **15** | **1641** | **15** | **1641** | **14** |
| Low-frequency antiparallel β-sheet | 1631 | 13 | 1631 | 12 | 1631 | 7 | 1631 | 8 | 1631 | 7 | 1631 | 9 | 1631 | 9 |
| **WL/Amyloid** | **1620** | **22** | **1618** | **16** | **1619** | **23** | **1619** | **25** | **1619** | **24** | **1619** | **25** | **1619** | **25** |
| Side-chains | 1606 | 4 | 1606 | 3 | 1606 | 4 | 1606 | 4 | 1606 | 4 | 1606 | 4 | 1606 | 5 |
| **60ºC, 16 days** | **PDZ3 ^a^** | | **Δ10ct-PDZ3** | | **E401R** | | **D332G** | | **D332P** | | **E334L** | | **E334Q** | |
|  | **Center (cm-1 )** | **Area (%)** | **Center (cm-1 )** | **Area (%)** | **Center (cm-1 )** | **Area (%)** | **Center (cm-1 )** | **Area (%)** | **Center (cm-1 )** | **Area (%)** | **Center (cm-1 )** | **Area (%)** | **Center (cm-1 )** | **Area**  **(%)** |
| β-turns | 1694 | 1 | 1694 | 4 | 1694 | 3 | 1694 | 2 | 1694 | 2 | 1695 | 1 | 1695 | 1 |
| High-frequency antiparallel β-sheet | 1680 | 10 | 1684 | 16 | 1683 | 14 | 1683 | 13 | 1683 | 11 | 1682 | 10 | 1681 | 10 |
| β-turns | 1668 | 11 | 1670 | 24 | 1669 | 23 | 1669 | 20 | 1669 | 20 | 1670 | 9 | 1670 | 7 |
| **Loops/Turns** | **1659** | **12** | **1659** | **8** | **1658** | **8** | **1658** | **9** | **1658** | **9** | **1659** | **15** | **1659** | **18** |
| α-helix | 1649 | 16 | 1650 | 11 | 1649 | 10 | 1650 | 9 | 1650 | 9 | 1648 | 13 | 1648 | 14 |
| **Flexible β-sheet/Random coil** | **1640** | **11** | **1641** | **11** | **1640** | **14** | **1641** | **15** | **1640** | **15** | **1640** | **10** | **1640** | **8** |
| Low-frequency antiparallel β-sheet | 1630 | 18 | 1631 | 8 | 1630 | 7 | 1631 | 6 | 1631 | 7 | 1631 | 14 | 1631 | 16 |
| **WL/Amyloid** | **1621** | **19** | **1621** | **17** | **1619** | **18** | **1619** | **22** | **1619** | **23** | **1619** | **25** | **1620** | **24** |
| Side-chains | 1607 | 3 | 1605 | 2 | 1607 | 4 | 1606 | 4 | 1606 | 4 | 1606 | 3 | 1606 | 3 |

^a^PDZ3 values are those already reported [Marin-Argany M, Candel AM, Murciano-Calles J, Martinez JC, Villegas S (2012) Biophys J 103: 738-747]. ^b^Δ10ct-PDZ3 values at 25ºC are those already reported [Murciano-Calles J, Martinez JC, Marin-Argany M, Villegas S, Cobos ES (2014) Biophys Chem 185: 1-7].
